# Phase field crystal models with applications to laser deposition: A review

**DOI:** 10.1063/4.0000226

**Published:** 2024-01-31

**Authors:** Duncan Burns, Nikolas Provatas, Martin Grant

**Affiliations:** Department of Physics, McGill University, Montréal, Québec H3A 2T8, Canada

## Abstract

In this article, we address the application of phase field crystal (PFC) theory, a hybrid atomistic-continuum approach, for modeling nanostructure kinetics encountered in laser deposition. We first provide an overview of the PFC methodology, highlighting recent advances to incorporate phononic and heat transport mechanisms. To simulate laser heating, energy is deposited onto a number of polycrystalline, two-dimensional samples through the application of initial stochastic fluctuations. We first demonstrate the ability of the model to simulate plasticity and recrystallization events that follow laser heating in the isothermal limit. Importantly, we also show that sufficient kinetic energy can cause voiding, which serves to suppress shock propagation. We subsequently employ a newly developed thermo-density PFC theory, coined thermal field crystal (TFC), to investigate laser heating of polycrystalline samples under non-isothermal conditions. We observe that the latent heat of transition associated with ordering can lead to long lasting metastable structures and defects, with a healing rate linked to the thermal diffusion. Finally, we illustrate that the lattice temperature simulated by the TFC model is in qualitative agreement with predictions of conventional electron–phonon two-temperature models. We expect that our new TFC formalism can be useful for predicting transient structures that result from rapid laser heating and re-solidification processes.

## I. INTRODUCTION

As energy is deposited onto a material, by means of a shock or laser, the system is driven out of equilibrium. The energy relaxation follows different minimizing pathways dependent upon the amount of heat input. For a crystalline solid, which exhibits a spatial lattice associated with a long-range correlation, the provided energy can cause the break of local symmetry. At low energies, phononic shock waves carry and dissipate the heat into the bulk. However, as the input fluence is increased, structural defects (dislocations, vacancy clusters) or phase transformations become another source for energy dissipation.

There has been recent interest to obtain fine material property control through energy deposition, for example, in the context of additive manufacturing.^1^ In the bulk solid state, laser pulsing excites the electrons. The electrons subsequently transfer their energy to the lattice, leading to shock peening effects,^2^ which promotes recrystallization and precipitation.^3–5^ Such behavior will be more pronounced near the material surface, where large energy differentials act as a driving force for such transformations. The effects of propagating shock fronts created by laser energy input are often associated with a thermal cycling response.^5,6^ Depending on input energy and corresponding residual stresses that develop, material failure may also ensue through melting, fracture, or gas entrapment process.^7^ To better exploit such emergent microscopic phenomena in materials, a deeper understanding of the energy dissipation pathways in irradiated materials is required.

Modeling laser-induced phononic shock propagation is challenging due to its inherently multi-scale nature. A lattice and electron temperature evolution equation is often used for a simplistic description of the resultant phonon release under laser irradiation.^8^ Substantial effort has gone into developing more complex two-temperature models (TTM).^9–11^ These methods are robust at very small time scales but often assume a uniform solid, averaging out the role of a microstructure such as interfaces, grain-boundaries, dislocation contributions, or metastable phases. Progress in modeling such processes requires methods that can evolve atomic structures on diffusional time scales.

Advances in high performance computing have made possible the simulation of kinetic theories to probe the complex material response and pattern formation mechanisms. For example, molecular dynamics methods are widely applied to the study of important atomic-scale phenomena occurring on short length and time scales, and in cases where an appropriate atomic interaction potential can be posited. However, such techniques can be computationally expensive to apply to the investigation of large polycrystalline networks on diffusional time scales. Here, spatially and temporally coarse grained field theories have found success. One modeling approach that retains and propagates information inherited from the atomic ordering in solids is the phase field crystal (PFC) model. The first such model was developed by Elder and Grant^12^ to track the evolution of an order parameter that approximated the temporally coarse-grained mass density distribution. The “PFC field” is evolved based on driving forces derived from a free energy functional designed to emulate the thermodynamic response of a material system to temperature, density change, pressure, and distortions. The PFC framework has seen quantitative and qualitative uses in studies of interfacial instabilities,^12–17^ defect evolution,^12,18–24^ and non-equilibrium (and equilibrium) phase transformations.^25–27^ Recent improvements heralding from solid-state hydrodynamics incorporate ballistic transport, which includes a time scale associated with dislocation energy emission, thereby allowing an extended description of dislocation and polycrystal relaxation.^16,28–30^ More recently still, the PFC density field has also been coupled to a temperature field to enable the study of energy transport associated with ballistic kinetics arising in non-equilibrium phase transformations and elasto-plasticity processes.^31^ The properties of the PFC methodology make it well suited to study the dissipation of phonon energy into these various defect structures on diffusional time scales.

In this work, we adopt the phase field crystal methodology to investigate nanostructure evolution mechanisms related to laser deposition in pure materials. We begin with an overview of the phase field crystal technique in the context of an early variant of the model, as well as a more recent one that allows for solid, liquid, and vapor phases. We then review dynamics available for both PFC models. We subsequently apply PFC modeling to the study of dissociative mechanisms relevant to laser processing. In particular, we investigate the isothermal structural response to noise induced lattice perturbations, highlighting the emergence of nano-voiding. We first examine a two-dimensional material, which approximates a material cross section perpendicular to the laser direction, and assume instantaneous energy diffusion through subsequent layers. We then add further complexity to our study by using a newly developed thermo-density coupled PFC model. We measure the average system temperature, finding a saturation response. Here, it is shown that lattice perturbations agglomerate, depositing energy at local regions in the system. The increased local energy deposits subsequently lead to metastable phase transformations, persisting until the temperature has equilibriated. Lattice excitations produced by a laser are approximated by the application of initial noise, which is a simplifying assumption. To confirm the inherent physics, we show a qualitative comparison of the lattice temperature predicted by our PFC model to that propagated by a two-temperature model of gold (Au). We conclude the paper by discussing the potential for the phase field crystal methods to be used for the investigation of nanostructure and microstructure evolution in polycrystalline materials subjected to laser heating.

## II. PHASE FIELD CRYSTAL (PFC)

Inspired by the classical density functional theory and continuum order parameter models, Elder and Grant developed the phase field crystal (PFC) methodology^12,32^ to investigate solidification. Herein, a time averaged continuum density field, *ρ*, is treated as a probability distribution associated with the atomic lattice and interstitial site occupancy. In a solid, the density field adopts a lattice structure set by the free energy, which will be discussed further below. Fluctuations in the density correspond to perturbation in the vacancy concentration (*c*) and hydrostatic strain ( ∇·u), which are tied by the relation

$$\delta \rho =-\delta c-\rho_{\text{ref}}\nabla \cdot u,$$ (1)

which is in accordance with the earlier work.^16^ In a homogeneous phase (i.e., liquid, gas, etc.), the density field is uniform and contains no spatial symmetries in contrast to the solid.

The recent success of phase field crystal methods for simulating the structural response lies in the simplicity of the model. The methodology can be broken down into three separate components for which there has been much development. First, the free energy construction, which specifies the equilibrium phases^25,26,33–41^ and associated thermodynamics.^13,26,31,32,36,37,42^ Second, the form of density propagation that describes the transition characteristics from one phase to another.^12,16,22,31,43–46^ Finally, the numeric implementation for the dynamic nanostructure modeling.^16,47–51^ This framework provides access to topological defect relaxation behaviors in a wide set of different materials, with characteristics that can be measured through strain and energy analyses (see, for example, Refs. 12, 16, and 52–54).

### A. PFC thermodynamics

As in traditional phase field models, the thermodynamic states of a PFC density field *ρ* herald from a free energy functional, *F*. In general, the free energy can be a highly complex function coupling many degrees of freedom together. Phase field crystal modeling relies on an expansion of the free energy around a reference homogeneous density ( $\rho_{\text{ref}}$) and temperature ( $T_{\text{ref}}$) at the liquid–solid transition point. This, in turn, provides a simplistic description of the transition free energy. We refer the interested reader to Refs. 32 and 55 for a derivation from the classical density functional theory. Performing such an expansion yields the following free energy functional:

$$\begin{array}{c} \tilde{F}=\frac{F}{T}=\rho_{\text{ref}}k_{B}\int_{\Omega }d^{2}r[A(T)+\lambda (T)\psi +\frac{\psi^{2}}{2}-\frac{\psi^{3}}{6}+\frac{\psi^{4}}{12}-\frac{\psi }{2}(C_{2}*\psi )+\cdots ], \end{array}$$ (2)

where $k_{B}T$ represents the energy scale and $\psi (r,t)=(\rho (r,t)-\rho_{\text{ref}})/\rho_{\text{ref}}$ denotes the dimensionless reduced density of the system. Interactions enter the theory through n-point direct correlation functions, *C_n_*, which differentiate solid and liquid phases. For simplistic lattice symmetries, it is often sufficient to truncate at the two-point correlation function. However, the higher order correlations are necessary with highly oriented phases (e.g., tetragonal lattice) or systems with large density differences.^27,36,37,41,56^ The * in Eq. (2) denotes a convolution operation, which reduces to a product when taken to Fourier space. The pre-factors *A*(*T*) and λ(T) depend on temperature *T* and are discussed further below. In principle, the expansion also yields pre-factors multiplying the $\psi^{2},\psi^{3},\psi^{4}$ terms.^15^ These factors are set to one here as their effect can be subsumed into the correlations and the other pre-factors.

**FIG. 1.**
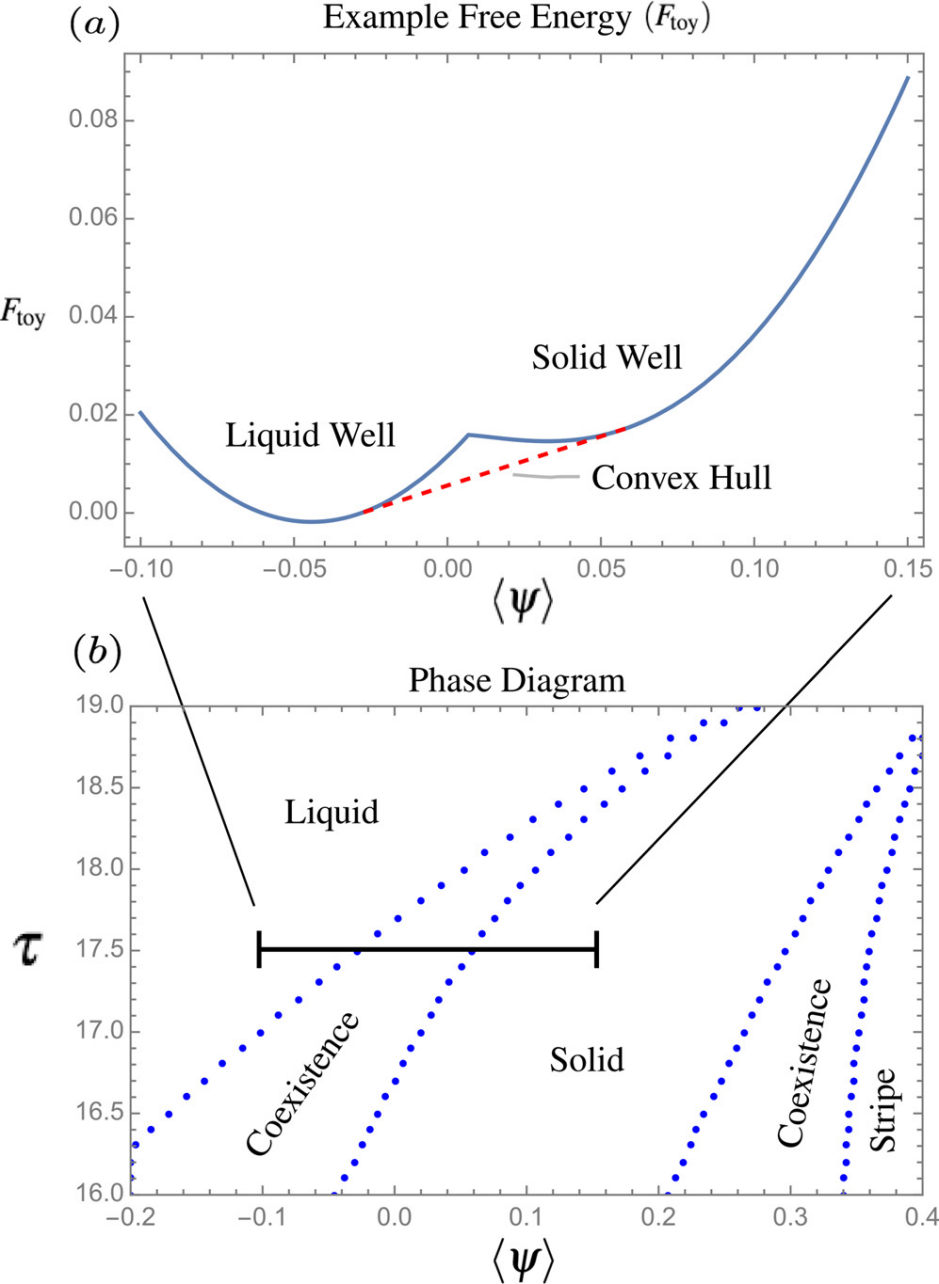
(a) A toy example of the free energy $\left(F_{\text{toy}}\right)$, which represents a rescaling of the true PFC free energy for τ=17.5. The red dotted line corresponds to a convex hull, across which are shown the tie-lines associated with the coexistence region for that temperature *τ*. (b) The phase diagram associated with the PFC free energy in Eq. (2) corresponding to the poly-PFC correlation used throughout this article.

It is convenient to analyze the solid-forming properties of Eq. (2) in Fourier space, where the spatial symmetries of the solid are reflected in a set of Bragg peaks at the reciprocal lattice vectors, $\left\{G_{j}\right\}$. The density of the solid is often assumed to have a one-mode ansatz

$$\psi (r,t)=\rho_{\text{ref}}\left(\bar{\psi }+\Sigma_{G_{j}}A_{\left|G_{j}\right|}e^{iG_{j}\cdot r}\right),$$ (3)

which yields an atomic density representation somewhat akin to transmission electron microscopy images of a solid. One may alternatively represent the density via a Gaussian approximation often used in density functional theories.^57^ The correlation function *C*_2_ can be construed to resonate with (be maximized at) the Bragg peak, which reinforces powers of the corresponding amplitudes ( $A_{\left|G_{j}\right|}$). To do so, one can fit *C*_2_ to the liquid state structure factor at or near the transition point.^12,58^ Typically,

$${\bar{C}}_{2}(k)=\{\begin{array}{cc} (1-B_{l}+2B_{x}k^{2}-B_{x}k^{4}+\cdots ), & \text{poly}-\text{PFC}\;\\ B_{l}+\sum_{\left\{G_{i}\right\}}B_{x}^{i}e^{-{\left(k-|G_{i}|\right)}^{2}/\sigma_{i}}, & X-\text{PFC}, \end{array}$$ (4)

where the polynomial-PFC is built as an expansion in gradients around the structure factor peak. The *B_l_* and *B_x_* parameters are temperature dependent reflecting the bulk moduli of the liquid and solid phase(s), respectively. In essence, the *B* parameters modify the location and the depth of the associated free energy wells, as in the example of Fig. 1. The polynomial form is often adopted when investigating simplistic 2D HCP and 3D BCC structures but requires either high-order expansions or the addition of other n-point correlations to account for different types of lattice symmetries. Meanwhile, the X-PFC methodology assumes Gaussian envelopes around the reciprocal lattice positions. Here, $B_{x}^{i}$ controls the peak height and *σ_i_*, the local peak curvature. Respectively, these contributions reflect the bulk and the metastable interfacial behaviors. We mention in passing that alternative peak structures, such as Lorentzians or Voigt functions, may be used. The change of the peak curvature modifies the effective liquid–solid interface properties.

Phase field crystal models rely on the design of the free energy to appropriately capture the thermodynamics of ordered or homogeneous phases. In doing so, one fits the bulk thermodynamic properties of each phase to existing measurements from the literature. Typical free energy fitting methods are described as by Elder *et al.*^32^ and Jaatinen *et al.*^42^ for fitting the equilibrium density of phases. In a recent paper, we also illustrated thermodynamic consistency equations for fitting the thermal expansion, heat capacity, and Gruneisen coefficients, thereby extending the thermodynamic relations that can be modeled quantitatively in the PFC approach.^31^ To do so, the parameters entering the free energy in Eq. (2) become temperature dependent.^31^ Thus, we may expand the parameters linearly as

$$A(\tau )=A_{0}+A_{1}\tau ,$$ (5a)

$$\lambda (\tau )=\lambda_{0}+\lambda_{1}\tau ,$$ (5b)

$$B_{l}(\tau )=-B_{l}^{0}+B_{l}^{1}\tau ,$$ (5c)

for which $\tau =T/T_{\text{ref}}$, with $T_{\text{ref}}$ denoting the reference temperature of the free energy expansion. These parameter expansions allow us to reproduce the aforementioned thermodynamics quantities found in databases such as by Dinsdale^59^ and Singh *et al.*^60^ The expansion is expected to be accurate over a wide temperature range for most metals but can be further expanded for more complex situations. It is noted that two different versions of thermal lattice expansion exist within the phase field crystal model. Previous attempts at incorporating a thermal lattice expansion have introduced a temperature dependent lattice periodicity.^61^ However, when simulating the phase field crystal model on a fixed volume simulation box, an isochoric ensemble is needed. The thermal expansion is thus captured in variations in the system pressure in accordance with thermodynamic relation, $\frac{\partial P}{\partial T}{|}_{V}=\alpha_{T}\kappa_{T}$, in which the partial derivative of pressure with respect to temperature is equal to the product of volumetric expansion coefficient multiplied by isothermal bulk modulus. Often, such variations in pressure appear as modulations of the local amplitude height, $A_{\left|G\right|}$, that reflects an increase in the interstitial occupancy probability (a form of vacancy density). This form of thermal expansion behavior has been extensively discussed in Ref. 31, where the temperature model is also developed. We note that a constant pressure ensemble can be investigated by simultaneously allowing the simulation volume to change.^27^ Here, the lattice expansion is expected to ensue without needing a temperature dependent $\left|G_{j}\right|$. An additional two sentences have been added to discuss the thermal expansion effect.

To investigate the parameter ranges of the model that will favor specific bulk phase, we build the associated phase diagram. Here, we solve the condition of phase coexistence encapsulates in the usual common tangent constructions (or Maxwell equal area construction), given by

$$\mu_{1}=\mu_{2},$$ (6a)

$$f_{1}-\bar{\psi_{1}}\mu_{1}=f_{2}-\bar{\psi_{2}}\mu_{2}.$$ (6b)

Here, $\mu_{i}=\partial F/\partial \psi$ is the chemical potential and $\bar{\psi_{i}}$ denotes the average density associated with phase *i* in coexistence. Although the analytic calculation of the coexistence relations is possible in the case of simple free energy forms, a more general approach applicable to any free energy form is obtained by making use of a convex hull algorithm of the free energy as detailed in Ref. 56. Repeating the calculation at different temperatures and average densities results in a phase diagram as illustrated in Fig. 1, where coexistence lines are determined when multiple phases are considered.

We can extend the free energy in Eq. (2) to account for additional phases, specifically, a vapor phase, which will play a key role when we investigate void formation and its interactions with other defects in materials. In the context of laser heating, voiding and fracture are prevalent, often destroying the sample of interest. Phase field crystal theories are typically expanded around a high temperature liquid, where differences between the liquid density and the associated solid coexistence density is negligible. To capture a vapor phase, we expand around the vapor coexistence density and introduce higher order correlations into the free energy to model the large density differences that exist between the vapor and liquid/solid. In this work, we adopt a simple modification of *F* introduced by Kocher *et al.* for this goal given by

$$\begin{array}{cc} F_{\text{vapor}} & =F+\rho_{\text{ref}}k_{b}T\int_{\Omega }d^{2}r\;[\left((aB_{l}+b)\frac{\psi_{\text{mf}}^{2}}{3}+c\frac{\psi_{\text{mf}}^{3}}{4}\right)\psi ]. \end{array}$$ (7)

Here, $\psi_{\text{mf}}$ is the effective mean field density, given by $\psi_{\text{mf}}=F^{-1}\left[e^{-k^{2}/\sigma }\hat{\psi }\right]$. Powers of $\psi_{\text{mf}}$ thus represent minimal, long-wavelength, higher order, correlation additions to the free energy. An upcoming work by Coelho *et al.*^41^ expands on this formalism by extending the powers of $\psi_{\text{mf}}$ to include both long *and* short wavelength correlations. The choice of coefficients *a*, *b*, and *c* adds a vapor well to the free energy and are tuned as in Ref. 15 to reproduce the effective phase diagram (see Fig. 5 for a crude reconstruction of an example phase diagram used in this work).

### B. Nanostructure evolution

This section will apply the above phase field crystal (PFC) formalism to the examination of phonon scattering in a nanocrystalline matter. From the perspective of models such as molecular dynamics or quantum density functional theory, there are no clear analytical or numerical approaches for up-scaling their behavior onto long-time scales relevant to the various transient processes controlling microstructure evolution, which in turn control most of the emergent properties of materials. Instead, a standard approach for capturing long-time relaxation processes is the use of Langevin type dynamics, which dissipate energy and evolve microstructure on time scales set by transport coefficients that enter the theory, the latter of which are determinable from short-time first principles models.

The most common type diffusional dynamics of *ψ* are governed by

$$\frac{\partial \psi }{\partial t}=\nabla \cdot D\nabla \frac{\delta F}{\delta \psi }+\nabla \cdot J_{\eta }.$$ (8)

These kinetics capture the local diffusion of mass and ordering, driven by gradients of the chemical potential,

$$\mu =\frac{\delta F}{\delta \psi }=k_{b}T\rho_{\text{ref}}[\lambda (T)+\psi -\psi^{2}/2+\psi^{3}/3-C_{2}*\psi ].$$ (9)

When the temperature is spatially dependent, one can expand or recast the expression in terms of linear and nonlinear operators acting on field *ψ* for computational simplicity. In this case, the leading order temperature factor is approximated via $T\approx T_{\text{ref}}$. The vector field $J_{\eta }$ denotes a noise current representative of fast processes on the order of the atomic vibrations, which have been averaged out when constructing the free energy functional and using it to drive the dynamics. The noise current is assumed to obey the fluctuation–dissipation theorem $\langle J_{\eta ,i}\rangle =0$ and $\left\langle J_{\eta ,i}J_{\eta ,j}\rangle =k_{b}TD\delta_{ij}\delta (t-t\prime )\delta (r-r\prime \right)$, where *δ_ij_* is the Kronecker delta between vector directions *i* and *j*. The diffusion, D, is often treated as a constant for simplicity, to allow for a linear description. In dynamic density functional theory, $D=D_{0}+D_{1}\psi$, to account for the fact that density cannot diffuse when at zero density. This addition may be important in the study of vapor phases, which achieve very low density.^17,27^ Since this work will deal mostly with the solid phase response, we approximate *D* as a constant. We mention in passing that Elder *et al.* had constructed an amplitude expanded version of the model, for which the diffusive Eq. (8) is assumed for a unit cell average density field and supplemented with energy-minimizing, non-conservative evolution equations for the amplitudes, $A_{\left|G\right|}$.^44^ In essence, by separating evolution equations, the approach prescribes a wave vector dependent diffusion coefficient. Further details for this formulation can be found in the review by Salvalaglio and Elder.^62^

**FIG. 2.**
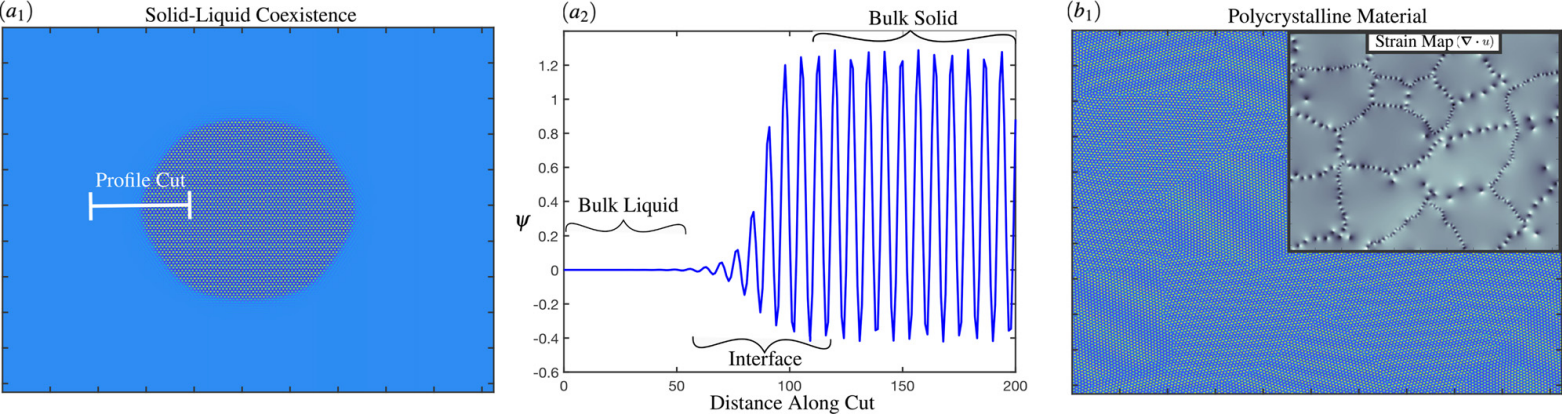
Illustrated examples of the phase field crystal methodology: ${(a}_{1})$ Solid–liquid coexistence. ${(a}_{2})$ The one-dimensional line profile highlighting bulk states and interfacial region. (*b*) Polycrystal featuring many orientations and grain boundaries. The inset shows the hydrostatic strain map with expression outlined in Ref. 16.

The dynamics of Eq. (8) propagate the density on diffusion time scales to relax metastable phases, defects, and interface structures. Equation (1) allows for defects to arise from fluctuations in the vacancy and strain fields. This further affects the relaxation of solid–liquid interfaces [Figs. 2(a_1_) and 2(a_2_)] or grain boundaries composed of dislocations [Fig. 2(b)]. Phase field crystal models driven by such diffusional dynamics have been used to investigate solidification theory (nucleation statistics,^63–65^ interfacial instabilities,^15,66^ and or the subsequent effect of solute in the case of multi-component materials^67^). Moreover, solid-state plasticity,^20^ grain-boundary coarsening,^14^ and precipitation^68^ can also be assessed.

Diffusional dynamics, such as those in Eq. (8), unrealistically tie the vacancy and the strain time scales together. Furthermore, the lack of momentum considerations implies that all structural response will be highly localized. Fluctuations in *ψ* couple both vacancy and strain flow. Both such contributions have been shown to be non-negligible for non-ideal crystals.^69,70^ Cohen *et al.* showed that it is necessary to consider transport characteristics arising from both the vacancies and strain in order to obtain a set of self-consistent hydrodynamic diffusion modes in solids.^71,72^ Within the confines of the diffusional dynamics outlined previously, the simultaneous evaluation of mechanical equilibrium can resolve some of the response discrepancies.^22,45,73^ Alternatively, the extension to hydrodynamic frameworks that explicitly incorporate velocity can be employed at the cost of an additional field.^74,75^ The hydrodynamic modes of a solid can also be incorporated into a single variable phase field crystal methodology.^16,28^ Specifically, it was shown there that the dynamics of Eq. (8) must be replaced by

$$\frac{\partial^{2}\psi }{\partial t^{2}}+\beta \left[\frac{\partial \psi }{\partial t}\right]=DT_{\text{ref}}\nabla^{2}\left(\frac{\delta F}{\delta \psi }\right)+\vec{\nabla }\cdot \vec{\eta },$$ (10)

where the addition of a second order time reflects the coupling of vacancy, strain, and momentum transport. As we have argued in the past, the *β* functional represents the vacancy diffusion and the phonon dissipation. Due to the phonon self-scattering and thermal scattering, different wavelength phonons have different decay characteristics. To account for such contributions, *β* can be expanded in powers of gradients,

$$\beta =\beta_{1}+\beta_{2}\nabla^{2},$$ (11)

with *β*_2_ setting the minimal propagating phonon wavelength.

The dynamics of Eq. (10), with $\beta_{2}=0$, were originally developed and used by Stefanovic *et al.* for the investigation of grain-boundary response to an externally imposed shear.^43^ It was demonstrated that the quasi-phonon disturbances produced by the shear, resulted in grain-boundary slip. Using the same model, Berry *et al.* later demonstrated the production of stacking faults in three-dimensional systems and quantitatively measured the dislocation climb and glide time scales.^20^ In a previous publication, we qualitatively demonstrated that two-time scale dynamics of our model in Eq. (10) can be used to tune the vacancy diffusion length and subsequently the growth morphology.^16^ As a consequence, the dynamics have a direct impact on the nucleation behavior as was found by Podmaniczky and Gránásy.^65^ Thereafter, we illustrated that two-dimensional polycrystalline materials subject to noise could melt and recrystallize.^24^ The addition of a non-zero *β*_2_, highlighted the contribution of short-range vibrations in the crossover region between ballistic and diffusional motion, emphasizing a characteristic Boson peak in the longitudinal phonon density of states.^76,77^ Of course, the lack of an explicit temperature contribution in the above dynamics assumes that propagating disturbances instantaneously deposit their energy across the entire sample, which acts as an infinitely large thermal bath.

**FIG. 3.**
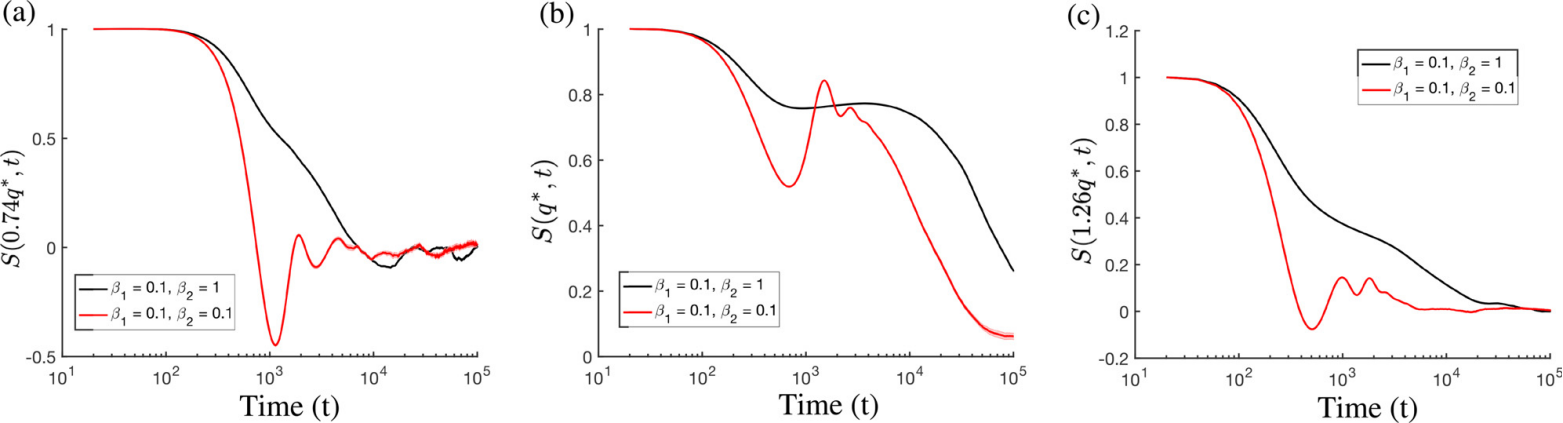
Plots of the density autocorrelation function [*S*(*q*, *t*)] as represented in Eq. (12) for phonon relaxation in a polycrystalline sample measured for three different wavevectors. The system corresponds physically to a solid whose atoms are highly excited through initial temperature fluctuations.

We applied Eq. (10) to the case of phonon scattering polycrystals in the isothermal limit.^24^ Results were compared against more short-time scale theories, such as molecular dynamics. The key measure used for this study is the autocorrelation function (or intermediate scattering function), defined by

$$S(q,t)=\left\langle \tilde{\rho }(q,0)\tilde{\rho }(-q,t)\right\rangle ,$$ (12)

which exhibits an exponential decay associated with each diffusion mode. We examined this metric in a polycrystalline system whose atoms are given an initial thermal agitation by perturbing their initial positions through the use of noise. The average of the autocorrelation function is performed over different initial conditions, iterations of noise, and angular dependencies. This metric is also readily obtainable experimentally through neutron scattering. Figure 3 shows the autocorrelation function results extracted from our simulations at three different wavevectors [(a) $q=0.76q^{*}$, (b) $q=q^{*}$, and (c) $q=1.26q^{*}$]. We note that not only should the temporal scaling of the autocorrelation be compared against faster time scale theories but also the wavevector dependent curvature. We suspect that through such a procedure, a suitable value for *β_i_* can be chosen, thereby shedding light on the physical time scale and dissipation introduced from temporally averaging our theory.

### C. Numerical methods

The dynamics encapsulated in Eqs. (8) and (10) need be propagated numerically. Since the theory is a set of partial differential equation, a wide variety of approaches is possible as long as appropriate choices of discretization parameters are used to ensure that the procedure is stable and convergent. Typically, one employs either generalized finite element analysis^49,78^ or semi-implicit Fourier methods^16,48^ dependent upon the type and required boundary conditions. One such technique used in this article is a newly developed pseudo-spectral method, whose details are described in Ref. 16. The key aspect of the procedure is that we recast the coupled dynamics into a linearized matrix form such as

$$\frac{\partial }{\partial t}\left(\begin{array}{c} \rho \\ \dot{\rho } \end{array}\right)=MP+Nl,$$ (13)

where M and *Nl* represent the matrix of linear operators and the vector of nonlinear factors, respectively. One may subsequently perform the exponential time integration. Due to the decoupling of Fourier modes, the associated matrix exponential can be computed analytically for fast time stepping. Due to the simplifications of operating in Fourier space, periodic boundary conditions are often imposed. When simulating large three-dimensional systems with many processors, one can use a recently developed fast Fourier transform procedure.^79^ We note that Dirichlet or Neumann conditions can be alternatively employed through the use of the appropriate trigonometric series. Additionally, more complex boundary conditions, such as traction boundary layers, can be weakly enforced through the addition of an external potential field.^43^ The form of Eq. (13) represents the matrix linearization of Eq. (10). In general, one can extend the vector *P* by the collection of all competing dynamical fields.

## III. LASER PROCESSING

**FIG. 4.**
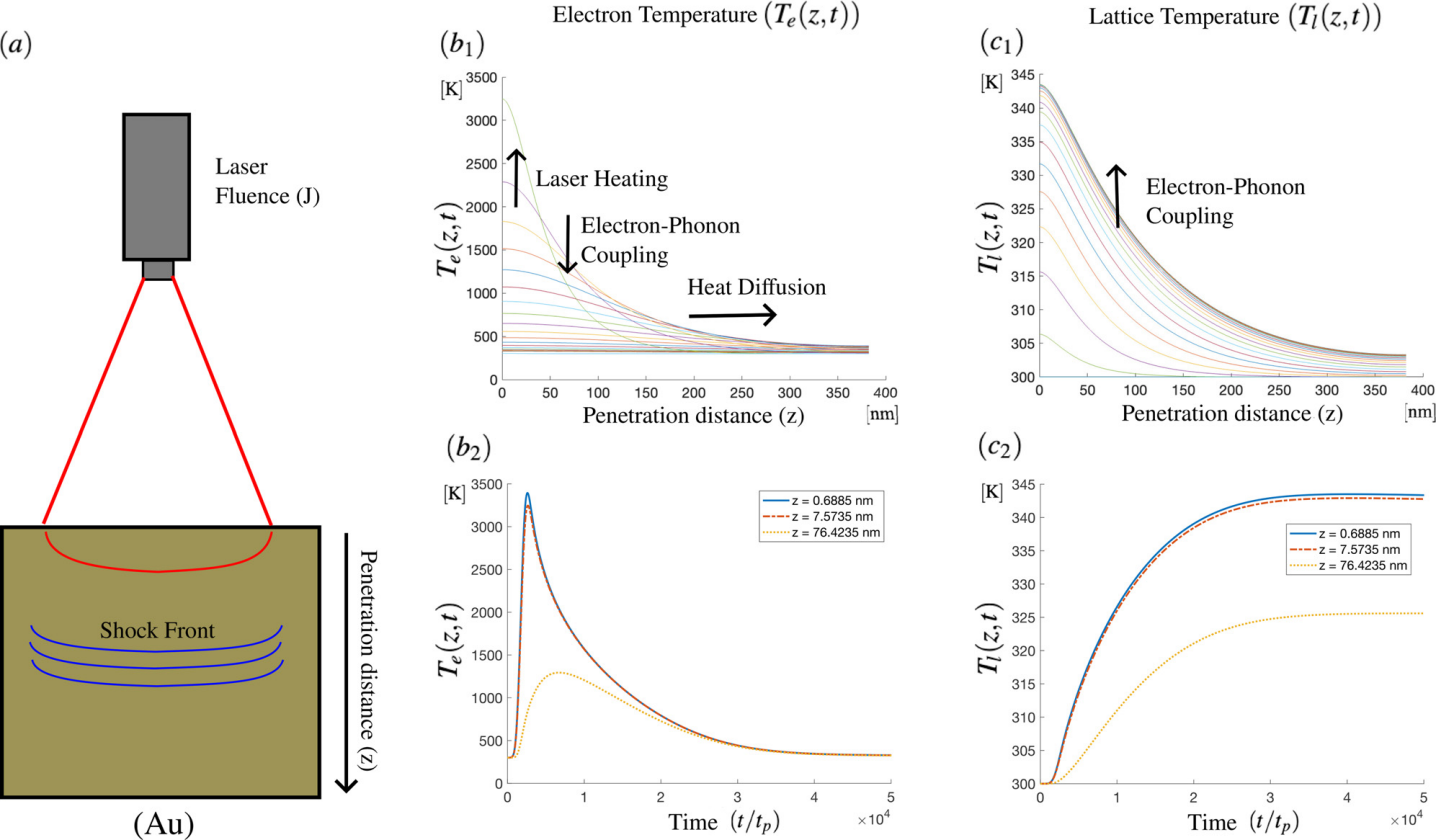
(a) A schematic of the processing conditions for the laser heating of a gold material. The properties of gold used are as listed in Ref. 8. Subpanels (b) and (c) plot the electron and the lattice temperatures simulated through Eq. (14).

Of current interest is the physics governing material response to laser irradiation. The high localized energies and energy gradients produced in such processes drive subsequent defect and metastable phase formation. The resultant nanostructure is of particular relevance for additive manufacturing and defect engineering. Herein, a laser deposits energy over some unit of area (fluence) through the excitation of electrons within a penetration depth associated with the skin-depth, *δ_z_*. The dissipation of the electron energy into the lattice structure results in propagating shock front that interacts with the global material structure. Figure 4(a) shows an example illustration of the process. Depending on the pulse time, *t_p_*, and the laser fluence, *J*, the lattice can reach temperatures favoring the ablation of the material. Even for reduced input energies, the local accumulation of energy around defect structures can also result in fracture, which is generally an unwanted by-product. We note that in the bulk shock fronts result in plastic deformation, while at interfaces, both phonon response and the laser-polarization couplings result in surface patterning. Structured induced by laser irradiation lies in a highly out-of-equilibrium regime, with a large number of cross-coupled time scales, as discussed further below.

### A. Two-temperature models

Theoretical models have been developed in the literature to understand the thermal distribution from the electron and the lattice in laser-irradiated solids. At the scale of the lattice temperature lag time, Qiu *et al.* developed the two-temperature model,

$$C_{e}(T_{e})\frac{\partial T_{e}}{\partial t}=\nabla \cdot (K_{e}\nabla T_{e})-G(T_{e}-T_{l})+Q(z,t),$$ (14a)

$$C_{l}(T_{l})\frac{\partial T_{l}}{\partial t}=\nabla \cdot (K_{l}\nabla T_{l})+G(T_{e}-T_{l}),$$ (14b)

which separates the evolution of an electron temperature (*T_e_*) from the lattice temperature (*T_l_*).^8^ Here, $C_{e(l)}$ and $K_{e(l)}$ denote the heat capacity and the thermal conductivity, respectively. The global relaxation of energy is achieved through a minimal electron–phonon coupling, *G*. The function,

$$Q(r,t)=\sqrt{4\;\text{log}\;2}\frac{(1-R)J}{\pi \delta_{z}t_{p}}e^{-\frac{z}{\delta_{z}}-4\;\text{log}\;2{\left(\frac{t-2t_{p}}{t_{p}}\right)}^{2}},$$ (15)

represents the absorbed energy from a laser pulse, with the material reflectivity, *R*. Using the coefficients for gold as outlined by Qiu and Tien in Ref. 8, we simulated the two-temperature model for a single pulse time of *t_p_* = 150 fs, with fluence, *J* = 200 $J/m^{2}$. As seen in Fig. 4(b), the electron temperature increases in response to the applied laser heating. Heat then diffuses while simultaneously exciting the lattice temperature [see Fig. 4(c)]. Once the electron and lattice temperatures are similar, the system equilibrates through thermal diffusion.

It is noteworthy that the two-temperature model has seen extensive use over the past three decades. Improvements to the theory have been suggested by the incorporation of additional time scales and accounting for more complex temperature dependencies of the model coefficients. However, in general, the theory assumes an ideal bulk solid. Alternative theories have also been postulated to account for phase transformation^10^ and elastic shock fronts.^80^ Such methods are macroscopic in character and only effectively account for dislocations and other nanoscopic features. To this end, it remains an open question on how to account for nanoscale plasticity in the context of laser processing.

### B. Noise-induced phase transformation

**FIG. 5.**
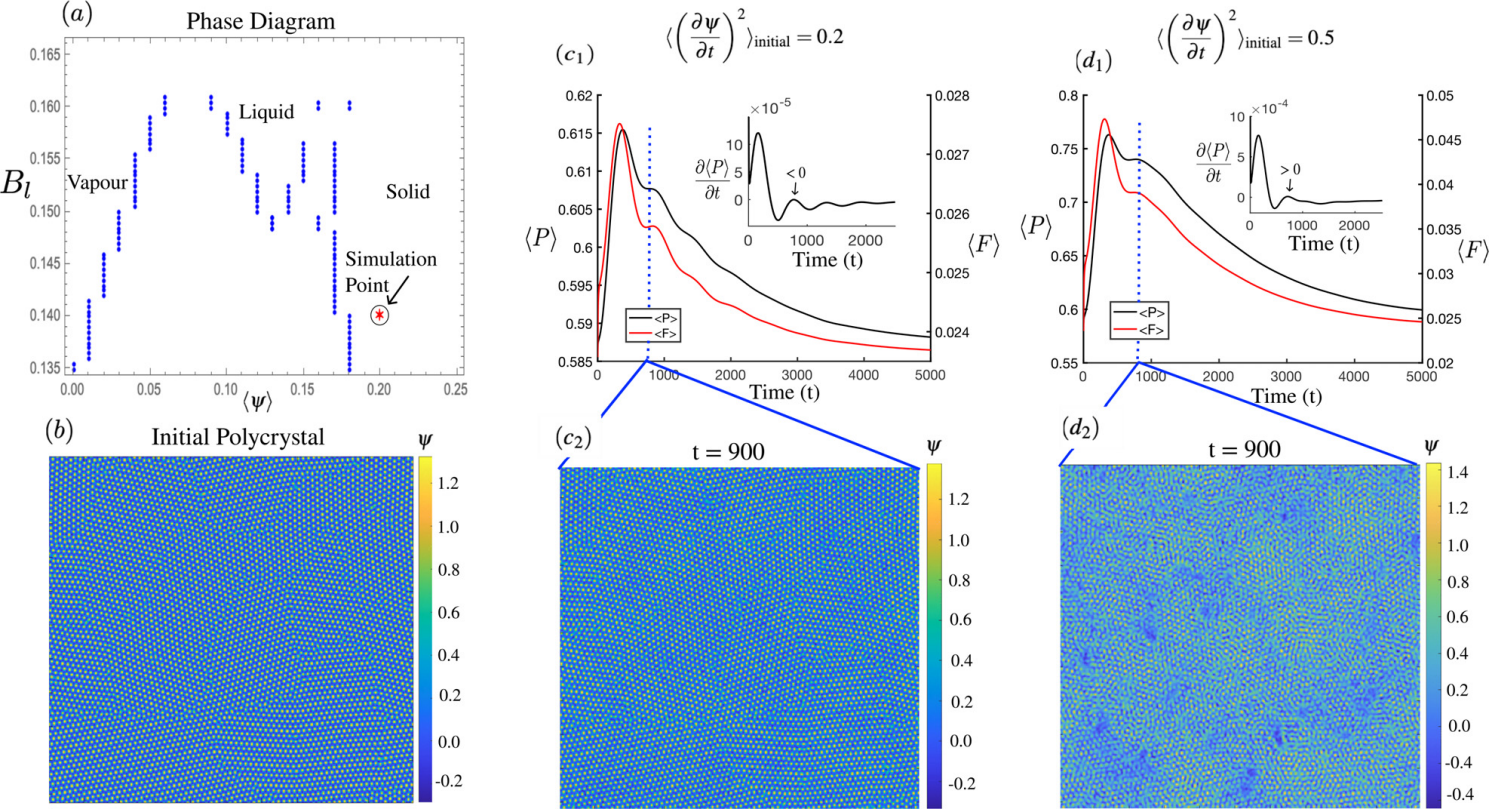
Subpanel (a) illustrates the solid–liquid–vapor phase diagram used, which is based on the free energy in Eq. (7). An initial polycrystal (b) is subjected to different magnitudes of an initial noise. The average system pressure, ⟨P⟩, and the average system free energy, ⟨F⟩ are shown in subpanels ${(c}_{1})$ and ${(d}_{1})$, which represent two different noise strengths. The insets highlight the slope of the average system pressure as a function of time. The corresponding density field, ψ(r), at t=900dt, is shown in subpanels (c_2_) and (d_2_).

It is instructive to examine if the two-time phase field crystal model that we have thus far discussed may serve as an extension of the two-temperature model that can capture important effects of nanoscale plasticity. We consider here two-dimensions, noting that such systems lack dislocation frustration mechanisms. Here, our two-dimensional system corresponds to a material cross section perpendicular to the laser direction. We simulated the response of a polycrystal [see Fig. 5(b)] to an initially applied noise magnitude. Following Ref. 24, the applied kinetic energy is given by

$$K_{l}(t)\propto {\left\langle {\left(\frac{\partial \psi }{\partial t}\right)}^{2}\right\rangle }_{\text{initial}},$$ (16)

where *K_l_* is proportional to the noise strength. For this, we utilize the vapor–liquid–solid PFC free energy of Eq. (7) with the dynamics of Eq. (10). The effective phase diagram of this model is illustrated in Fig. 5(a). In response to the initial perturbation, phonons travel through the material, collecting at sites of grain boundaries. The phonon propagation can be observed by the oscillatory nature of the average system pressure as in Fig. 5(c). At increased energies, metastable voids (vapor pockets) are nucleated at grain-boundary sites. The deposition of phonon energies that causes the voiding results in a suppression of further phonon motion as seen in Fig. 5(d). Although not explicitly shown in Fig. 5, we also observe two-step phase transition at higher effective *B_l_* values for which the stability of the liquid phase is increased. The applied noise is to be viewed as a simplification of a shock front passing through our system. The simulations may thus shed light on material rolling processes, adaptive manufacturing, and shock peening, albeit in an isothermal limit. It is noted that in higher spatial dimensions, the dislocations can be frustrating, thereby forming amorphous-like phases.^81^ Furthermore, the shock-induced formation of unstable void phases described herein is a potential mechanism for fracture and metastable phases/precipitates formation found in additively manufactured metals.^6^

### C. Heat transport effects; thermal field crystal (TFC)

The noise-induced phase transformations of Sec. III B were performed in isothermal conditions, which treats the system as being in contact with a heat sink, instantaneously relaxing temperature gradients. However, in laser processing applications, the system does not have a chance to equilibrate the free energy. Subsequently, the temperature gradients produced by the laser, and latent heat release, can drive the system to form various metastable structures in the context of the average system temperature. To examine this effect, we employ a recently developed extension to the phase field crystal methodology that coupled density to temperature. Here, the dynamics of Eq. (10) are supplemented by the heat transport equation

$$\begin{array}{c} \left[-T\frac{\delta^{2}F}{\delta T^{2}}\right]\frac{\partial T}{\partial t}=\left(h-\mu +T\frac{\partial \mu }{\partial T}-\frac{D_{T}}{D_{v}}\right)\frac{\partial \psi }{\partial t}\\ +\frac{D_{T}^{2}}{D_{v}}\left(\frac{\nabla T\cdot \nabla T}{T^{2}}-\frac{\nabla^{2}T}{T}\right)\\ +\alpha_{T}\nabla^{2}T+S(\vec{r},t), \end{array}$$ (17)

which propagates a continuum temperature field through driving forces derived from the phase field crystal free energy. The variables, *h*, *D_T_*, *D_v_*, and *α_T_* refer to the enthalpy, thermodiffusion coefficient, vacancy diffusion coefficient, and thermal conductivity, respectively. The function $S(\vec{r},t)$ denotes an externally applied heat source, such as a heat sink. In comparison to previous thermo-density phase field crystal formulations,^46,61^ Eq. (17) additionally supports ballistic transport, thereby allowing use in conjunction with either PFC density equation of Sec. II B.

**FIG. 6.**
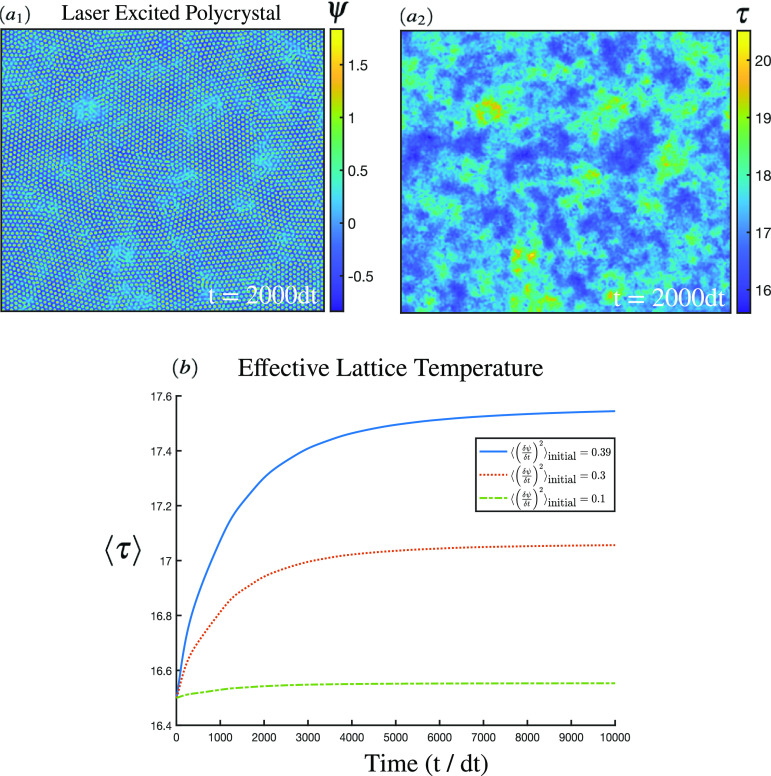
A polycrystal is prepared using the solid–liquid free energy with the corresponding phase diagram shown in Fig. 1(b). The system is subjected to an initial instance of conserved noise and allowed to relax through the TFC dynamics of Eqs. (10) and (17). The density field and the dimensionless temperature field are illustrated at t=2000dt in subpanels (a_1_) and (a_2_), respectively. Subpanels (a_1_) and (a_2_) correspond to a high initial noise of $\left\langle {\left(\delta \psi /\delta t\right)}^{2}\right\rangle =0.39$. The time dependence of the average system temperature for three noise strengths is shown in subpanel (b).

Figure 6 illustrates a TFC simulation of laser heating. Subpanel (a_1_) highlights the density field profile with the metastable liquid formed after a polycrystal is subjected to an initial noise of $\left\langle {\left(\frac{\partial \psi }{\partial t}\right)}^{2}\right\rangle =0.39$. At locations where the perturbation energy accumulates locally, metastable liquid pools form. We observe in the temperature profile shown in subpanel (a_2_) that the defects have high local temperatures. In this case, the deposited laser energy exceeds the melting point, leading to liquid pools, which subsequently re-solidify at rates dictated by the thermal diffusion. The average system temperature for three different input energies is shown in subpanel (b). The dominant characteristic is the saturation of the temperature input through the initial noise perturbation. Only the $\left\langle {\left(\frac{\partial \psi }{\partial t}\right)}^{2}\right\rangle =0.39$ case exhibited substantial melting. The melting transition referred to above leads to subtle curvature changes as heat is absorbed (noticeable around t=1000dt). In addition, during late stage resolidification, latent heat release causes an increase in the temperature.

We may now compare the predicted lattice temperature profile predicted by the thermal phase field model to the two-temperature model for laser deposition. In the latter case, we observe a generic saturation curve as illustrated in Fig. 5(c2). During the sub-picosecond ( $t/t_{p}<1$) regime, there is an exponential ramp up in the lattice temperature as heat flows from the excited electrons. At late stages, the temperature at a given penetration depth decreases as the temperature diffuses through the bulk. The results of Fig. 6(b) show the same qualitative saturation curve as the late-time lattice temperature results of Fig. 5. We note that the electron excitation time scale lies below the numeric time step of the TFC methodology. As a consequence, no early stage ramp up can be observed from the PFC model. Nonetheless, one can, in principle, directly solve the electron temperature equation simultaneously with the TFC equations. In this case, it remains to be determined how the electron–phonon coupling constant is modified for polycrystalline materials. The late stage response of the two models is thus similar and also includes important latent heat effects that can shift the temperature curvature. Although a crude energy deposition approximation was used here, we have illustrated that the salient thermal physics of lattice energy transport are captured by the PFC formalism while additionally also accounting for the plastic deformation mechanisms in polycrystalline materials. The TFC methodology, with the possible extension to include the electron temperature dynamics, may thus serve as a future means of accessing the nanostructure dynamics of laser processing.

## IV. CONCLUSION

In this article, we illustrated the application of the phase field crystal methodology for the investigation of nanostructure response in laser deposition. We first provided an overview of the phase field crystal technique. We then applied the theory to laser processing. In particular, we investigated the isothermal structural response to noise-induced lattice perturbations, observing nano-voiding, when a vapor phase was taken into account. We further investigated the athermal regime using a newly developed thermo-density kinetic coupling. We measured the average system temperature, finding a late-time saturation temperature response. Here, lattice perturbations agglomerate, depositing energies in local regions. The increased energy in these zones subsequently causes local melting or transformations into metastable structures, which persist until the temperature has equilibrated. Finally, we compare the lattice temperature predicted by a two-temperature model of gold (Au) to our TFC simulations, finding a similar late-time temperature response. To this end, we have shown that phase field crystal methods can be used to study nanostructure and microstructure response of laser heating.

Numerous future directions are accessible to the PFC modeling techniques reviewed in this work. One may employ multiferroic phase field crystal theories to explicitly account for the polarization, magnetization, and subsequently laser coupling.^82^ Such an investigation may shed light on the high spatial frequency laser-induced periodic surface structure formation, which are the subject of the current research.^83–85^ Alternatively, the laser can be modeled through solving an additional electron temperature, though it remains to be determined the appropriate electron–phonon coupling near defect sites. By extending the theory to a multi-component material, one can investigate the concomitant precipitation formation pathways. The fine control of the precipitation fractions can further lead to regulating the bulk material properties. Finally, in the two-dimensional simulations discussed throughout this article, dislocation structures are prone to anneal and do not experience the frustration of three-dimensional systems. Future work may thus entail tabulating differences between the recrystallization kinetics in two vs three-dimensional systems. In particular, we suspect that high energy may allow the formation of highly frustrated dislocation-mediated structures to emerge. The plethora of future avenues that are accessible to length and time scales of phase field crystal theories may permit more detailed understanding of laser induced microstructure formation in rapid solidification.

## Data Availability

The data that support the findings of this study are available from the corresponding author upon reasonable request.
